# Biological Role of *Trichoderma harzianum*-Derived Platelet-Activating Factor Acetylhydrolase (PAF-AH) on Stress Response and Antagonism

**DOI:** 10.1371/journal.pone.0100367

**Published:** 2014-06-25

**Authors:** Chuanjin Yu, Lili Fan, Qiong Wu, Kehe Fu, Shigang Gao, Meng Wang, Jinxin Gao, Yaqian Li, Jie Chen

**Affiliations:** 1 Department of Resource and Environmental Science, School of Agriculture and Biology, Shanghai Jiao tong University, Shanghai, P. R. China; 2 State Key Laboratory of Microbial Metabolism, Shanghai Jiao Tong University, Shanghai, P. R. China; Louisiana State University, United States of America

## Abstract

We investigated the properties of platelet-activating factor acetylhydrolase (PAF-AH) derived from *Trichoderma harzianum*. The enzyme, comprised of 572 amino acids, shares high homology with PAF-AH proteins from *T. koningii* and other microbial species. The optimum enzymatic activity of PAF-AH occurred at pH 6 in the absence of Ca^2+^ and it localized in the cytoplasm, and we observed the upregulation of PAF-AH expression in response to carbon starvation and strong heat shock. Furthermore, PAF-AH knockout transformant growth occurred more slowly than wild type cells and over-expression strains grown in SM medium at 37°C and 42°C. In addition, PAF-AH expression significantly increased under a series of maize root induction assay. Eicosanoic acid and ergosterol levels decreased in the PAF-AH knockouts compared to wild type cells, as revealed by GC/MS analysis. We also determined stress responses mediated by PAF-AH were related to proteins HEX1, Cu/Zn superoxide dismutase, and cytochrome c. Finally, PAF-AH exhibited antagonistic activity against *Rhizoctonia solani* in plate confrontation assays. Our results indicate PAF-AH may play an important role in *T. harzianum* stress response and antagonism under diverse environmental conditions.

## Introduction

The platelet-activating factor acetylhydrolase (PAF-AH) belongs to the phospholipase A_2_ (PLA_2_) enzyme superfamily. These enzymes hydrolyze ester bonds at the *sn*-2 position of glycerophospholipids to release free fatty acids and lysophospholipids, which often trigger signal transduction pathways in animal or human cells. For example, arachidonic acid is the precursor of eicosanoids, prostaglandins, and leukotrienes, all of which play important roles in the inflammatory response [1]–[3]. Specifically, PAF-AH is involved in human health and disease by promoting proinflammation [4], [5] and mediating stress responses in eukaryotic cells. Kono, et al. [6] reported intracellular type II PAF-AH could protect against oxidative stress-induced hepatic injury by metabolizing oxidized phospholipids. Furthermore, the PAF-AH gene in *Saccharomyces cerevisiae* significantly enhances the yeast's resistance to oxidative stress [7].

*Trichoderma*, which is present in diverse habitats, has been used as a biocontrol agent for many decades [8]–[10] and has maintained stress-related gene expression (*e.g., hsp70* and *hex1*) to adapt to varied environments [11], [12]. This beneficial fungal genus antagonizes other fungi by producing antibiotics and extracellular enzymes or by outcompeting other species for nutrients [13]–[15]. Many important antagonism-associated genes or proteins are expressed during mycoparasitic interactions of *Trichoderma* with its host, such as chitinase, glucanase, and PAF-AH [16], [17]. In previous studies, we screened many *Trichoderma koningii* (T30) transformants generated by restriction enzyme-mediated integration under different stress conditions and found that PAF-AH transformants were susceptible to H_2_O_2_ and other stresses. We currently observed *Trichoderma harzianum* (T28) PAF-AH expression was significantly upregulated under heat stimulus and that T28 PAF-AH transformants grew more slowly than the wild type strain. These results indicate PAF-AH plays an important role in *Trichoderma* stress response, but little information is available regarding its physiological function(s). Therefore, in this work, we analyzed the PAF-AH gene derived from *Trichoderma harzianum* and investigated its function in stress response and antagonism.

## Materials and Methods

### Strains and culture conditions

The wild type strain *T. harzianum* T28 (CCTCC AF 2013026) was stored in the Laboratory of Resource and Environmental Science. Wild type and transformants were maintained in PDA (Difco, Becton Dickinson & Co., USA) at 28°C until sporulation occurred. *Escherichia coli* DH5α and BL21 (DE3) were purchased from Invitrogen (Carlsbad, CA, USA) for plasmid propagation and protein expression, respectively. Bacterial strains were grown in Luria-Bertani (LB) broth or LB agar plates supplemented with kanamycin sulfate (0.1 mg/mL) or ampicillin (0.1 mg/mL).

### Generating PAF-AH deletion and over-expression transformants

A genome walking kit (Takara Bio, Kyoto, Japan) was used to clone the PAF-AH gene, resulting in a transformant referred to as P6 [18]. Primers used in the genome walking assay are listed in Table S1. Total cDNA of T28 was synthesized with the PrimeScript RT Reagent Kit (Takara Bio) and used as template for PAF-AH ORF amplification with primers PAF-AH-F/PAF-AH-R. Amplicons were ligated to the pMD-18-T Simple Vector (Takara Bio) and sequenced (BioSune Company, Shanghai, China). To generate PAF-AH deletion transformants, the homologous recombination cassette was constructed, and the 824 bp 5′ and 804 bp 3′ flanking sequences of PAF-AH were cloned with Ko-up-F/Ko-up-R (5′ region) and Ko-down-F/Ko-down-R (3′ region). The cloned flanking sequences were restriction digested with *Hind*III, *Xba*I, *Kpn*I, or *Sac*I (Ferments, Canada), respectively, and gel purified and ligated to modified plasmid pCAMBIA1300 (provided by Chu Long Zhang of Zhejiang University). *Agrobacterium tumefaciens*-mediated transformation was performed as previously described [19], [20] for the generation of PAF-AH deletion and over-expression transformants. The *trpC* promoter and terminator sequences from the vector pSilent-1 [21] were used to construct the PAF-AH ORF over-expression cassette.

The genomic DNA of transformants were isolated by a modified CTAB method [22]. In order to identify the PAF-AH deletion transformants: (1) the *hyg* gene (for hygromycin B resistance) was amplified with primers Hyg-F/Hyg-R; (2) the PAF-AH deletion was verified by attempting to amplify PAF-AH; (3) T-DNA insertion numbers were determined by using primers RB-F/Hyg-R; and (4) primers PAF-AH-UP-F and Hyg-R were used to confirm the *hyg* cassette position. In addition, 40 µg genomic DNA from T28 and transformants was digested with *Hind*III and *Bam*HI (Ferments, Canada). Finally, Southern blotting was carried out to confirm the transformants as described previously [19]. Similarly, over-expression transformants were identified with primers Hyg-F/Hyg-R by PCR to confirm that T-DNA inserted into the genome of T28. We used primers *TrpC*-F/Terminator-R to amplify the PAF-AH ORF over-expression cassette, and Southern blotting was again used to determine the copies of PAF-AH over expression cassette present. Over-expression PAF-AH transformant (OE1) strains were identified with one T-DNA insertion site (Fig. S1).

### Prokaryotic expression and enzyme activity assay

The PAF-AH gene was amplified by PCR and inserted into vector pET-28a (+) (Novagen, USA), and the construct was transformed *into E. coli* BL21 (DE3). Gene expression was induced with addition of 1 mM IPTG. Transformants were collected, suspended in lysis buffer (50 mM NaH_2_PO_4_, 300 mM NaCl, 10 mM Imidazole), and subjected to sonication. The solution was centrifuged at 5000 rpm for 10 min, and resulting supernatants were transferred to Ni-NTA agarose columns (Qiagen, Germany), and PAF-AH enzymatic activity was analyzed by the PAF-AH assay kit (Cayman, USA). For immunoblotting assays, protein samples were separated by SDS-PAGE and transferred to a nitrocellulose membrane. After electroblotting, filters were saturated with 5% non-fat dry milk in TBS with 0.1% Tween for 1 h at room temperature. Monoclonal 6×His-tag PAF-AH-specific antibodies (1∶8000 dilution, Sigma-Aldrich) were used as primary antibodies, and an anti-mouse peroxidase conjugate (1∶9000 dilution, Sigma-Aldrich) used as secondary antibody for detection of PAF-AH. For chemiluminescence detection, ECL Plus Western Blot detection reagent (GE Healthcare) was used.

### Subcellular localization of PAF-AH

For *in vivo* fluorescence analysis, knockout (KO40) transformants expressing PAF-AH C-terminally fused with GFP under pdg promoter [23] was constructed. Microscopic observations were performed using a Leica DM2500 fluorescent microscope with emissions detected at 495–530 nm. Micrographs were observed at 1000× magnification.

### Protein extraction, 2D gel electrophoresis, and protein identification

One hundred microliters conidial suspension (1×10^6^ mL^−1^) from wild type T28 and transformant strain KO40 were added to a 500 mL sterile flask with 200 mL SM medium [24] in triplicate. The solution was incubated in a rotary shaker (180 rpm) at 28°C for 6 days. After incubation, mycelia were filtered and thoroughly washed with sterile ddH_2_O. The mycelia were ground by mortar and pestle with liquid nitrogen, and TCA-acetone was used to extract the mycelial proteome. Total protein concentrations were determined by the Bradford method [25] with bovine serum albumin (Bio-Rad) as a protein standard. Two-dimensional gel electrophoresis (2-DE) was performed according to Bio-Rad standard protocols with slight modifications. The total protein of T28 and KO40 (300 µg each) was resolved in 125 µL rehydration buffer (8 M urea, 2 M thiourea, 2% CHAPS, 100 mM DTT, and 1% IPG 3–10 buffer) and was loaded onto 7 cm Immobiline DryStrips (pH 3–10, linear; Bio-Rad, USA) for rehydration. The running program of isoelectric focus was 250 V slow for 1 h, 1000 V fast for 1 h, 4000 V line for 4 h, 32,000 V/h, and 500 V fast for arbitrary times. The IPG strips were treated in equilibration buffer I (6 M urea, 0.375 M Tris-HCl [pH 8.8], 20% glycerol, 2% SDS, and 2% DTT) for 15 min followed by equilibration buffer II (6 M urea, 0.375 M Tris-HCl [pH 8.8], 20% glycerol, 2% SDS, 2.5% iodoacetamide) for another 15 min. The equilibrated IPG strips were placed on top of a vertical 12% polyacrylamide gel (0.5 mm thick) covered with 0.5% low-melting agarose and 25 mM Tris-HCl (pH 6.8). Each gel was run at 5 mA for 1 h and 15 mA for 1 h and then stained by Coomassie Brilliant Blue [26]. Gels were observed with a Versdoc 3000 scanner (Bio-Rad) and analyzed using PDQuest software (version 7.1, Bio-Rad, Hercules, CA, USA). Statistically significant changes in spot intensity (Student's *t*-test at *P*<0.05) indicated significant differential expression of PAF-AH. Gel analysis and protein identification were performed at the research laboratory of Fudan University.

### Quantitative real-time PCR

Total cDNA was synthesized from 1 µg wild type or other transformants' RNA using the PrimeScript RT Reagent kit with gDNA Eraser (Takara, Japan). Quantitative real-time PCR (qRT-PCR) was performed using a SuperReal PreMix (SYBR Green) kit (TIANGEN, China) with the synthesized cDNA as template. The PCR conditions were as follows: 95°C for 30 s for initial denaturation and 40 cycles each consisting of 95°C for 20 s, 58°C for 30 s, and 72°C for 20 s. For this experiment, an FTC-3000 Real-Time PCR System (Funglyn Biotech, Canada) was used according to the manufacturer's instructions. The qRT-PCR was carried out with primers Actin-F and Actin-R for amplifying the reference control *actin* gene. Expression levels of genes *hex1* (Accession no. KF356403), *cu/zn superoxide dismutase* (*sod*) (Accession no. JX481779), and *cytochrome c* (Accession no. JX481780) from strains T28 and KO40 were tested using the following primer sets: hex1-F/hex1-R, cu/zn sod-F/cu/zn sod-R, and cytochrome c-F/cytochrome c-R (Table S1), respectively. The qRT-PCR assay was carried out in three biological replicates with every reaction in triplicate, and the gene expressions level was determined using the 2^−ΔΔCT^ method as previously described [27]. The efficiency of primers is shown in Tables S2 and S3.

### GC/MS analysis of fatty acids and sterols

Conidial suspensions (100 µL of 1×10^6^ conidia mL^−1^) from wild type T28 and transformant KO40 were incubated in 200 mL SM medium at 28°C with 180 rpm shaking for 6 days. The mycelia were filtered and washed with sterile ddH_2_O three times and then lyophilized for 3 h. The mycelia (0.5 g) were ground by mortar and pestle with liquid nitrogen, and fatty acids were extracted by the standard chloroform/methanol method. After saponification in methanol with 1 M NaOH in a 75°C water bath for 30 min, the fatty acid methyl ester was dissolved in 1 mL normal hexane. The fatty acids and sterols were analyzed by GC/MS according to the previously described method [28] with slight modifications. Approximately 1.5 µL sample was injected into a 7890A–5975C gas chromatograph (Agilent, USA), and chromatographic separation was conducted on a DB-5MS column (30 m×0.25 mm×0.25 µm). Finally, mass spectra were acquired from *m*/*z* 33 to 500 at full scan, and the acceleration voltage was initiated after a solvent delay of 3 min. The data were then compared with reference spectra from the NIST 2011 library.

### Antagonism and stress plate agar assays and other methods

Plate confrontation assays were performed as described formerly [16], [29]. Pathogens were grown on agar plates previously colonized by wild type or transformant *T. harzianum* strains with a cellophane membrane in order to detect antagonistic activity by *T. harzianum* secreted factors. To test *Trichoderma* resistance to distinctive types of abiotic stresses, 7-mm-diameter SM plugs of T28 and transformants were placed at the center of agar plates containing variable pH SM medium or SM medium supplemented with different concentrations of H_2_O_2_. Plates were incubated at 28°C for 24 h. In parallel, strains were incubated at 37°C, 40°C, or 50°C for 24 h to evaluate *T. harzianum* thermotolerance.

Protein sequences were aligned using the CLUSTAL W method and the Phylogenetic neighbor-joining (NJ) tree was constructed with the MEGA 5.1 software. SOD and catalase activity assay were carried out with the same amount of tissue proteins that dissolved in 0.1 M PBS, and the strains were cultured the same as in 2-DE protein extraction. Data were measured with Microplate Reader.

### Statistical analysis of experimental data

All experiments were performed in independent biological triplicates. Data were analyzed statistically using SAS 8.0 software (significance indicated by *P*≤0.05). Graphs were prepared with GraphPad Prism 5.

## Results

### Cloning the PAF-AH gene from *T. harzianum* T28

**Figure 1:**
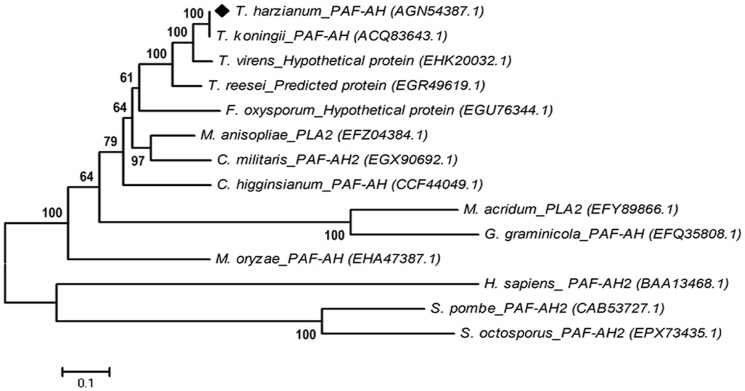
Phylogenetic neighbor-joining (NJ) tree of the PAF-AH protein sequences. The phylogenetic NJ tree was generated with MEGA 5.1 software from alignments of protein sequences obtained using CLUSTAL W. The PAF-AH protein from *T. harzianum* T28 (marked with a black square) and related proteins of representative strains from the GenBank database are shown.

The cDNA of PAF-AH (Accession no. JX481778) was amplified from synthesized T28 cDNA. The 1783 bp gene contains 65 bp of intronic sequence and a 1719 bp ORF that encodes 572 amino acids. A neighbor-joining tree was constructed after aligning PAF-AH protein sequences with those from different organisms, including other *Trichoderma* spp. (teleomorphic *Hypocea* spp.), yeast, and humans (Fig. 1) to infer the evolutionary relationships among PAF-AH. PLA_2_ Proteins from PLA_2_ family were distributed into two main groups according to their phylogenetic relationships. The PAF-AH of *T. harzianum* T28 was located with other ascomycete sequences, and it formed a distinct subclade with a PAF-AH protein from *T. koningii*, supported by a bootstrap value of 100%.

### Prokaryotic expression of PAF-AH and analysis of its biochemical characteristics

**Figure 2:**
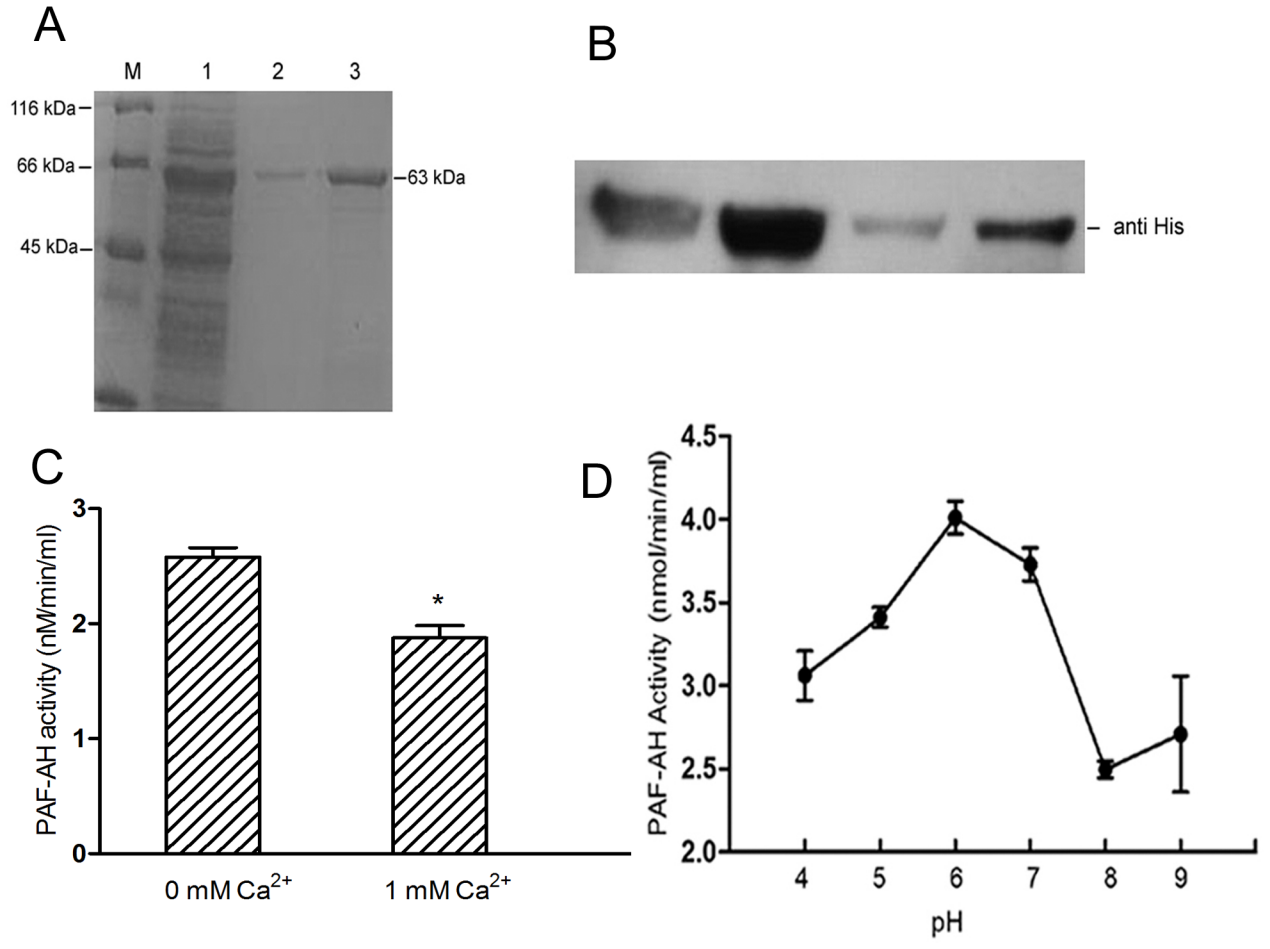
Properties of the PAF-AH enzyme. (A) The protein was expressed in *E. coli* BL21 (DE3). M, protein marker. 1, supernatant of induced lysis cells; 2 and 3, purified PAF-AH from the elution buffer. (B) Immunoblot analysis of purified PAF-AH using anti-His tag antibodies as indicated. (C) The PAF-AH activity assay experiment was carried out as described in Materials and Methods. Error bars denote SD. Asterisks indicate significant differences in enzyme activity under the condition of no Ca^2+^ compared with 1 mM Ca^2+^ (Student's *t*-test, *P*<0.05). (D) The PAF-AH activity assay was conducted under different pH conditions.

The PAF-AH ORF was digested with restriction enzymes *Bam*HI and *Eco*RI and then ligated with plasmid pET-28a. The presence of pET-28a-PAF-AH was verified by PCR and DNA sequencing. Then, pET-28a-PAF-AH with N-terminal fusions and metal-binding 6× His tag was transformed into *E. coli* strain BL21 (DE3), to which 1 mM IPTG was added for induction of gene expression. A soluble fusion protein was used for SDS-PAGE and Western blot analysis (Fig. 2A and Fig. 2B), and we identified the recombinant protein as a band of 63 kDa, which we confirmed by immunoassay.

The PAF-AH enzymatic assay demonstrated PAF-AH activity was significantly inhibited by adding 1 mM Ca^2+^ to the cultures. The highest activity of PAF-AH was observed at pH 6 in the absence of Ca^2+^ (Fig. 2C and 2D).

### Construction of PAF-AH KO deletion transformants

**Figure 3:**
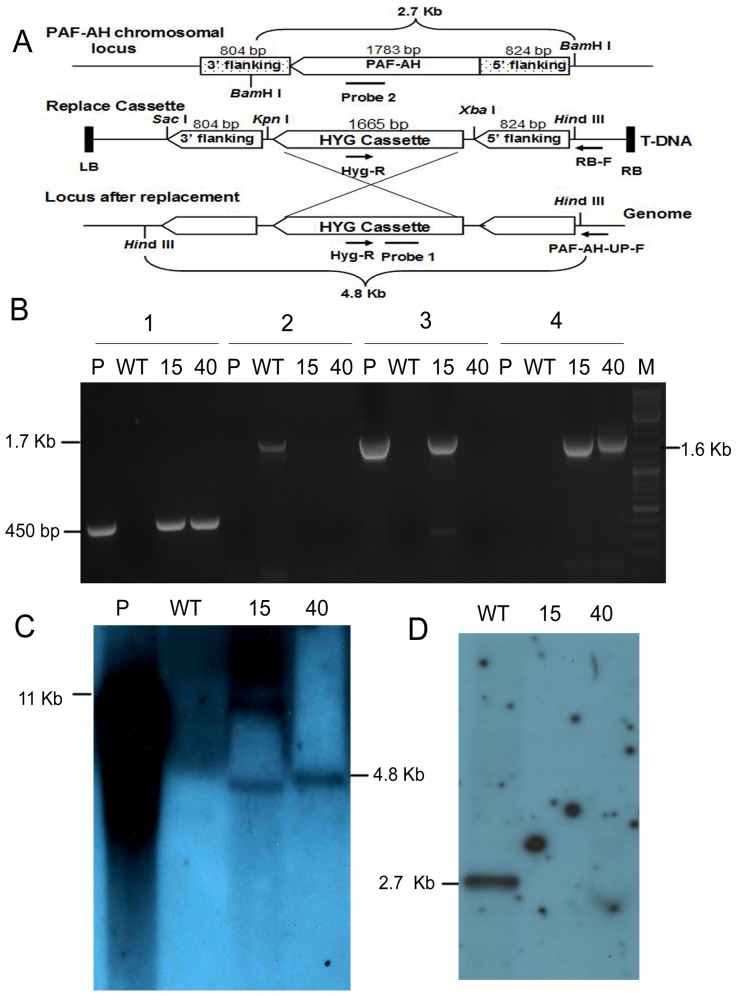
Screening of PAF-AH knockout transformants. (A) Physical map of homologous recombination. (B) Identification of transformants by PCR: 1, fragment of the hygromycin B resistance gene (*hyg*); 2, amplified fragment of PAF-AH from genomic DNA; 3, amplified T-DNA fragments adjusted to the right border of plasmid; 4, fragment amplified with primers PAF-AH-UP-F and Hyg-R. (C) Southern blot to verify T-DNA insertion copies of knockout transformants. The 450 bp fragment amplified from the *hyg* gene was labeled with ^32^P-dCTP as probe 1. (D) Southern blot to confirm PAF-AH was replaced using the 500 bp fragment of PAF-AH as probe 2. WT, wild type strain; P, plasmid; KO15 and KO40: PAF-AH knockout transformants; M: DNA marker.

Strains of PAF-AH deletion (KO) transformants were generated to investigate the PAF-AH gene's function in *T. harzianum* T28. The modified vector pCAMBIA1300 was used to replace the PAF-AH gene with the gene for hygromycin B resistance (Fig. 3A). We used four primers to screen the ideal transformants from 50 PAF-AH gene knockout candidates, and knockouts were detected by PCR (Fig. 3B). The *hyg* cassette was successfully amplified in KO15 and KO40 transformants, while the PAF-AH ORF was not. The flanking fragment adjacent to the right border was present not only in plasmid, but also in transformant KO15, which meant there was likely another T-DNA insertion site in the transformant. Fortunately, the additional insertion was absent in transformant KO40. Additional PCR confirmed that the *hyg* cassette was inserted into the correct position in both transformants.

Next, Southern blot analysis confirmed the PAF-AH gene had been replaced (Fig. 3D). However, while using the *hyg* gene as a probe, transformant KO15 showed another T-DNA insertion compared with transformant KO40 (Fig. 3C). In addition to genomic DNA analysis, we verified the transformant at the transcriptional level (Fig. S2). We also tested PAF-AH activity in T28, KO40, and OE1 (over-expression PAF-AH) strains and, as expected, found the lowest or highest PAF-AH activity in KO40 or OE1, respectively (Fig. S3). These results suggested transformant KO40 was the most acceptable knockout clone for further PAF-AH studies.

### PAF-AH subcellular localization

**Figure 4:**
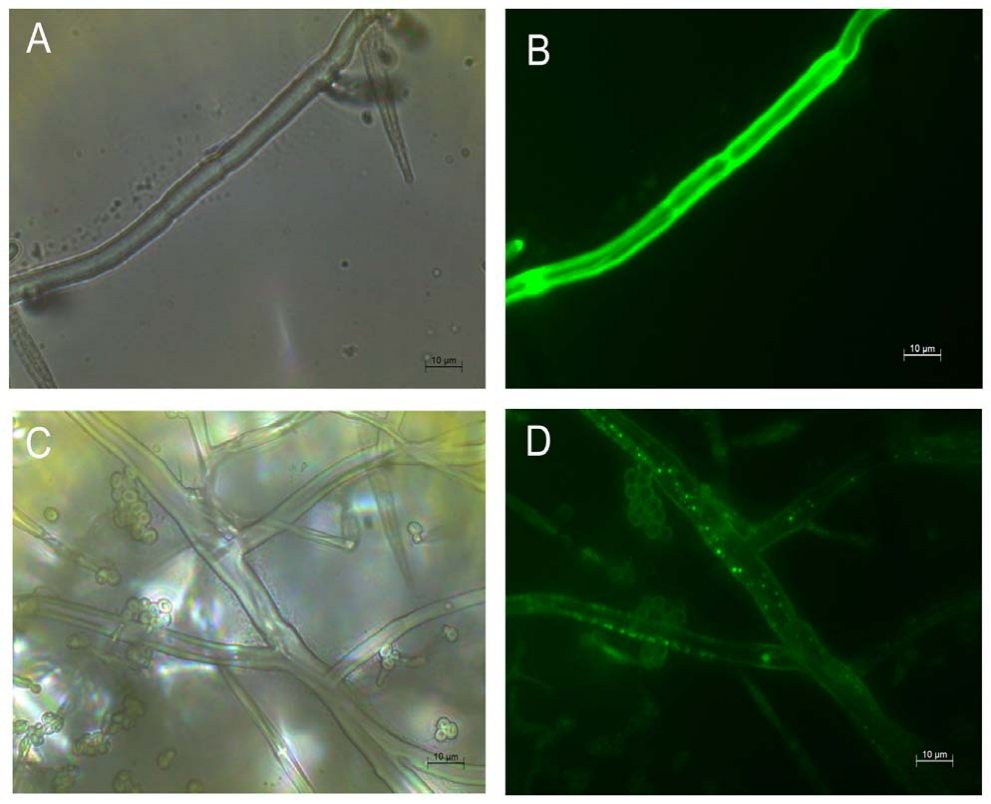
Subcellular localization of PAF-AH. (A) Micrographs of the positive control. (B) Fluorescent images of the positive control. (C) Micrographs of the PAF-AH protein-fused eGFP. (D) Fluorescent images of PAF-AH-fused eGFP. The eGFP was fused with the C terminus of the protein. Scale bars represent 10 µm.

To observe localization of the PAF-AH protein, PAF-AH C-terminally fused eGFP was transformed into KO40. The Pdg promoter + eGFP set was transformed into the KO40 as the positive control. The fluorescence of the positive control was distributed throughout the cells (Fig. 4B), but fluorescence was only observed in the cytoplasm of the KO40 transformant cells carrying the PAF-AH + eGFP fusion (Fig. 4D), indicating PAF-AH is localized in the cells' cytoplasm.

### PAF-AH-mediated response to various stress factors

**Figure 5:**
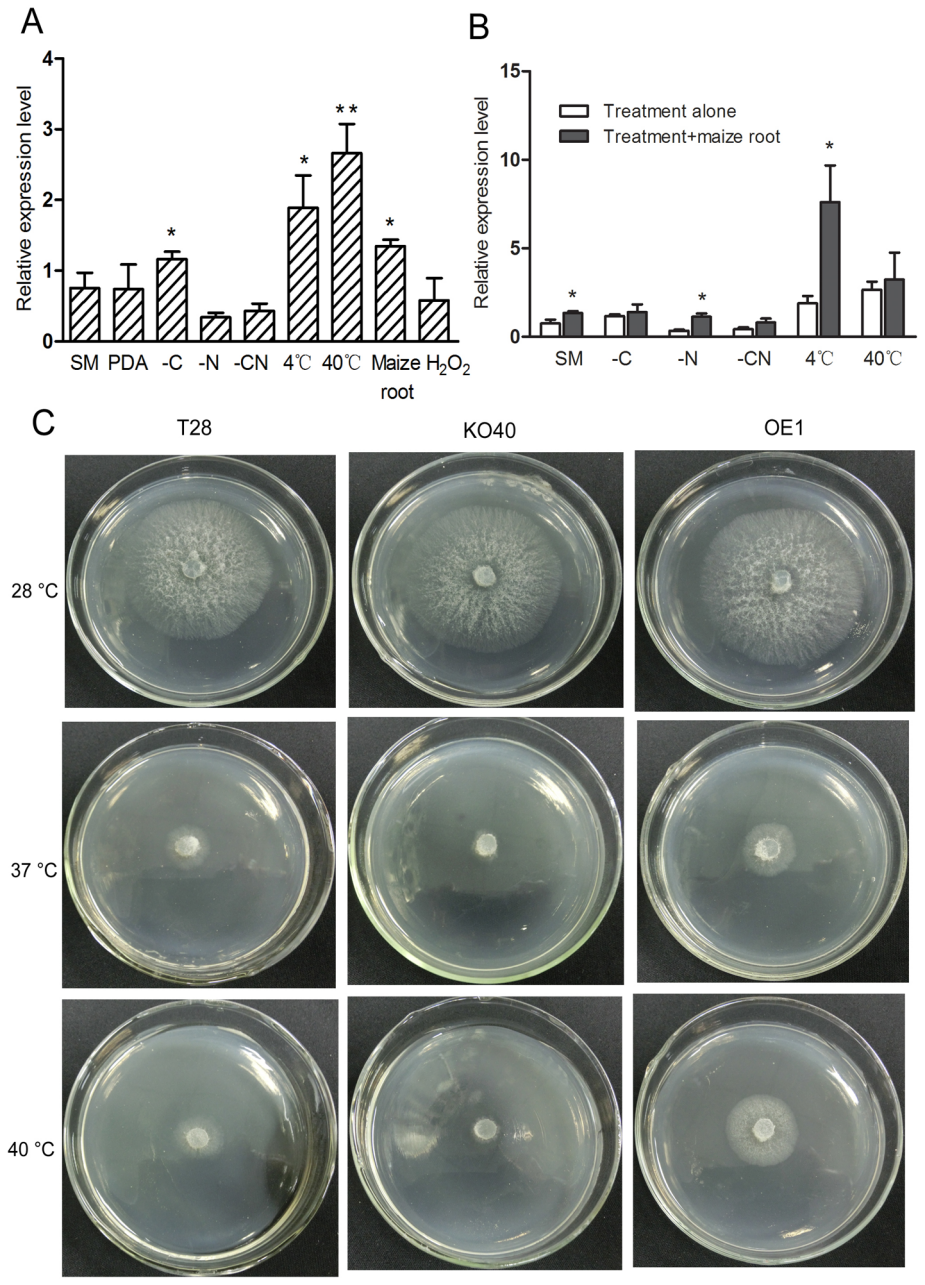
Quantitative real-time PCR shows PAF-AH mRNA expression levels. (A) Identical volumes (1×10^6^) of spores were cultured in SM liquid medium for 72 h, and mycelia were washed once with sterile ddH_2_O, transferred to fresh medium, (100 mL), and grown with shaking at 180 rpm, at 28°C for 4 h unless otherwise stated below. The following conditions were tested: SM medium, PDA, SM medium without carbon source (-C), SM medium without nitrogen source (-N), SM medium without carbon and nitrogen sources (-CN), 4°C in SM medium, 40°C in SM medium, 1% maize root in SM, and 5 mM H_2_O_2_ in SM. (B) PAF-AH expression levels in SM, -C, -N, -CN at 4°C and 40°C or all above stress condition with 1% maize root induction. The actin gene was used as the internal control. The experiment was carried out in three biological repeats. * indicated a significant difference compared with SM medium (Student's *t*-test, *P*≤0.05), and * * indicated *P*≤0.01. Results shown are means ± SD (n = 3). (C) Different strains grew on SM plate agar at different temperatures. The photograph was taken after 24 h incubation at each temperature. T28, wild type; KO40, PAF-AH knockout transformant; OE1, PAF-AH over-expression transformant.

The wild type strain T28 was cultured in SM medium at different conditions for further analyzes of PAF-AH activity. We analyzed PAF-AH mRNA expression levels in response to a series of induced conditions using qRT-PCR, and data showed PAF-AH was strongly upregulated in response to heat shock at 40°C, carbon starvation, and cold stimulus (4°C). Moreover, levels of PAF-AH expression were also upregulated after exposure to maize inbred line (Huangzhou 4) roots (Fig. 5A). Furthermore, to investigate the mechanism of PAF-AH expression induced by maize roots, we subsequently tested the mRNA expression level of PAF-AH by qRT-PCR at different stress conditions. The results demonstrated PAF-AH was upregulated by induced maize roots without nitrogen source (-N) and at 4°C (Fig. 5B). Because the expression of PAF-AH was strongly upregulated by induced heat shock (40°C), we performed a series of plate agar assays testing thermotolerance at different temperatures (Fig. 5C). Among the strains tested on SM agar at 37°C and 40°C, KO40 showed the lowest growth, and OE1 exhibited the highest growth level, suggesting PAF-AH promotes thermotolerance in *T. harzianum*.

### Analysis of fatty acids and sterols by GC/MS

Differences between fatty acid and sterol profiles in the transformants and wild type strains were analyzed with GC/MS. Ten distinct types of fatty acids and ergosterol were found and are shown in Table 1. The peak area showed the proportion of each fatty acid or ergosterol present in the sample. Data indicate the peak area percentages of tetradecanoic acid, pentadecanoic acid, hexadecanoic acid, eicosanoic acid, and ergosterol declined in the transformants when compared to the wild type. Interestingly, eicosanoic acid and ergosterol were significantly lower in the transformant (*P*<0.05), indicating they are typically associated with PAF-AH expression.

**Table 1:** GC/MS analysis of differences among fatty acids and sterols regulated by PAF-AH in *T. harzianum*.

No.	RT^a^	Formula	Match^b^	Chemical Name	Peak area of T28^c^	Peak area of KO40^c^	*T*-test (*p*)
1	12.695	C14:0	915	Tetradecanoic acid	0.3069%	0.2891%	0.5558
2	13.746	C15:0	956	Pentadecanoic acid	0.5039%	0.4668%	0.5447
3	14.787	C16:1	964	9-Hexadecenoic acid (Z)	0.5095%	0.5297%	0.3051
4	15.116	C16:0	957	Hexadecanoic acid	23.5281%	20.7071%	0.1226
5	16.714	C17:0	933	Heptadecanoic acid	0.2974%	0.4062%	0.3722
6	18.134	C18:2	967	9,12-Octadecenoic acid (Z,Z)	34.8427%	37.3763%	0.2855
7	18.277	C18:1	960	11-Octadecenoic acid	26.8206%	26.9661%	0.7460
8	18.737	C18:0	954	stearic acid	7.2698%	7.2987%	0.8259
9	23.695	C20:0	875	Eicosanoic acid	0.0848%	0.0653%	0.0497
10	35.99	C24:0	849	Tetracosanoic acid	0.2612%	0.3161%	0.1982
11	49.387	C28H44O2	835	Ergosterol	2.0640%	1.8576%	0.0461

Note: a, RT, retention time of specific fatty acid; b, match scores >800 meant the result was definite; c, the percentage of each fatty acid and sterols in total peak area meant that the difference was significant (Student's *t*-test, *P*<0.05).

### Differential gene and protein expression mediated by PAF-AH

**Figure 6:**
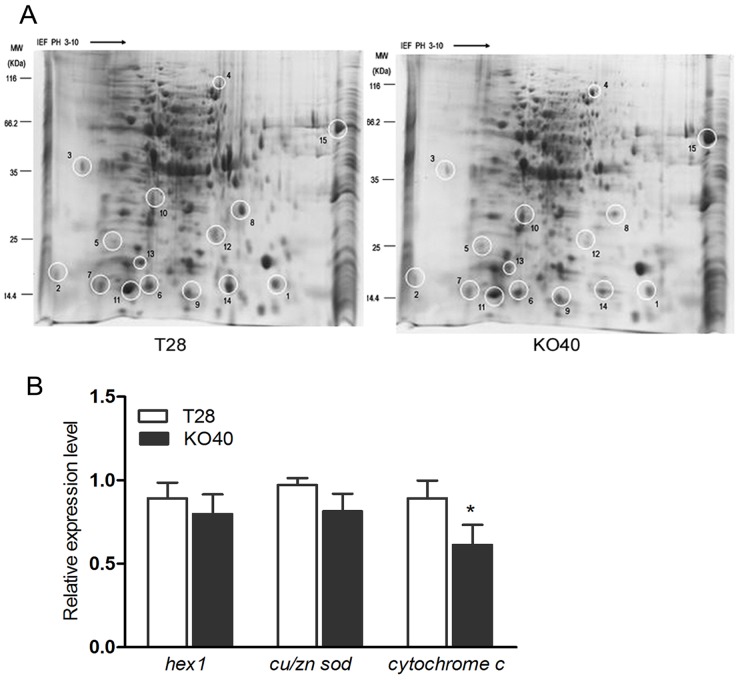
Gene and protein expression of PAF-AH in strains T28 and KO40. (A) 2-DE profiles of T28 and KO40 and qRT-PCR expression levels of *hex1*, *cu/zn sod*, and *cytochrome c* (B) 2-DE gels of total proteins were subjected to isoelectric focusing on IPG strips (7 cm, pH 3–10) and then separated in the second dimension on 12% polyacrylamide SDS gels. Gene expression was evaluated by qRT-PCR with the actin gene as the internal control. The experiment was carried out in three biological repeats. * indicated a significant difference (Student's *t*-test, *P*≤0.05). Results shown are means ± SD (n = 3).

Two-dimensional gel electrophoresis was performed to compare differential proteins expression between T28 and transformant KO40, and 15 differential proteins were identified (Fig. 6A). Among them, expression levels of nine proteins were downregulated and six were upregulated in the KO40 transformant. Cytochrome c, peptidyl-prolyl *cis-trans* isomerase, hypothetical protein CHGG_10818, HEX1, and Cu/Zn superoxide dismutase, as well as seven unknown proteins were identified by MS/MS (Table 2). Further analysis revealed that expression of cytochrome c, HEX1, and Cu/Zn superoxide dismutase was downregulated in the KO40 transformant, indicating PAF-AH may be closely involved in upregulating the above three proteins. We confirmed the mRNA expression relationship of *hex1*, *cu/zn sod*, and *cytochrome* c corresponded at the protein level with qRT-PCR, which showed the expression of *hex1*, *cu/zn sod*, and *cytochrome c* was indeed lower in transformant KO40 than in the wild type (Fig. 6B).

**Table 2:** Identification of differentially expressed proteins from *Trichoderma harzianum* T28 in response to PAF-AH

Spot No.^a^	Accession No.^b^	Protein species	Mr/PI	Peptides matched	Score	Protein description	Changes^c^
1	gi∣219564039	*Fungal sp. M160*	734.4778/7.94	9	57	RNA polymerase II second largest subunit	↓
2	gi∣291195753	*Magnaporthe oryzae*	1137.6232/9.25	6	103	Cytochrome C	↓
3	gi∣119399388	*Aspergillus clavatus NRRL 1*	734.4819//8.55	12	59	Conidiophore development protein HymA	↓
4	gi∣295629366	*Tuber melanosporum*	870.4828/11.32	9	60	Unnamed protein product	↑
5	gi∣310795078	*Glomerella graminicola M1.001*	868.4838/6.69	7	48	4-hydroxybenzoate polyprenyl transferase	↑
6	gi∣156841824	*Vanderwaltozyma polyspora DSM 70294*	850.4662/6.27	15	47	Hypothetical protein Kpol_1030p35	↑
7	gi∣169779443	*Aspergillus oryzae RIB40*	737.2981/5.89	6	132	Peptidyl-prolyl cis-trans isomerase	↓
8	gi∣304557446	*Hypocrea lixii*	942.4566/6.59	6	451	HEX1	↓
9	gi∣226294971	*Paracoccidioides brasiliensis Pb18*	868.5631/6.04	10	50	Conserved hypothetical protein	↑
10	gi∣83415459	*Hyaloraphidium curvatum*	735.4395/11.71	16	55	RNA polymerase II second largest subunit	↑
11	gi∣88175532	*Chaetomium globosum CBS 148.51*	713.4163/10.45	12	77	Hypothetical protein CHGG_10818	↓
12	gi∣304557446	*Hypocrea lixii*	786.3860/6.59	17	387	HEX1	↓
13	gi∣119466929	*Hypocrea lixii*	1940.0492/5.58	2	113	Cu/Zn superoxide dismutase	↓
14	gi∣302925511	*Nectria haematococca mpVI 77-13-4*	953.5060/5.27	6	67	Predicted protein	↓
15	gi∣88177387	*Chaetomium globosum CBS 148.51*	750.5027/9.08	7	51	Hypothetical proteinCHGG_08869	↑

Note: a, the numbering corresponds to the 2-DE gel in Figure 6A; b, accession number according to Mascot Search Results; c, “↓” meant “downregulated” and “↑” meant “upregulated”.

### Role of PAF-AH in antagonism and mycoparasitic interactions

**Figure 7:**
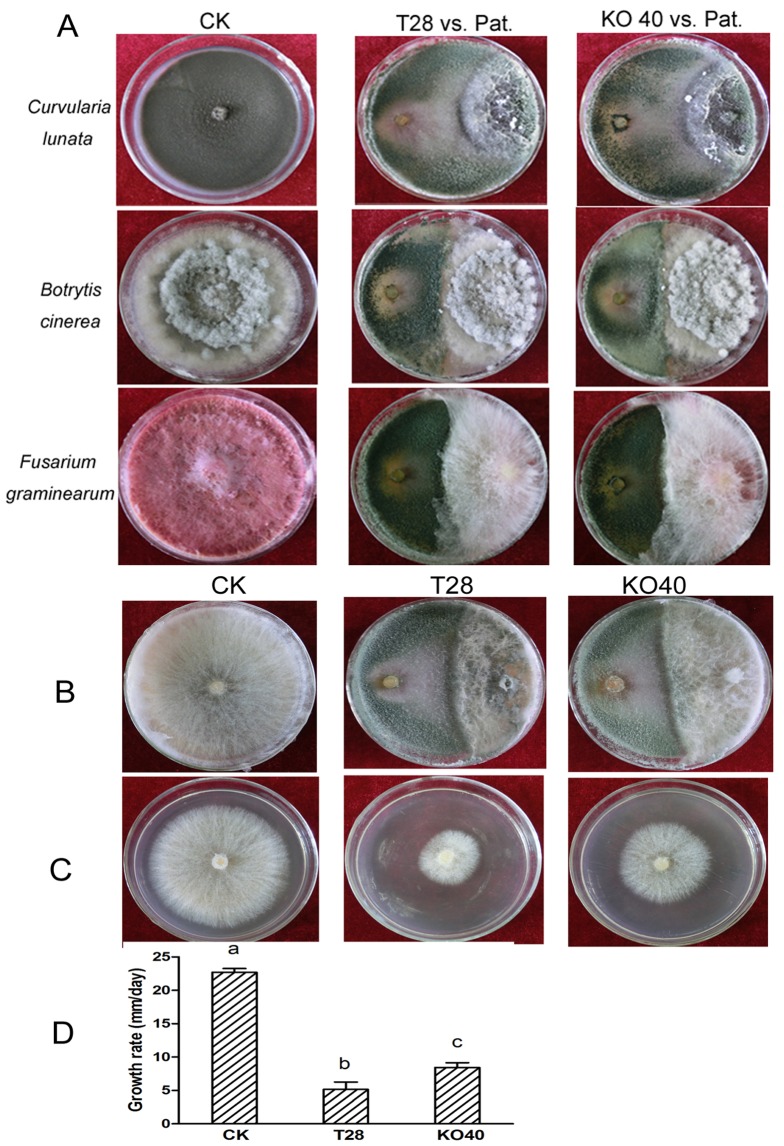
Plate confrontation assays of T28 and KO40 strains. (A) Confrontation experiments between T28 or KO40 strains against three different pathogens ( = Pat.). (B) Plate confrontation against *R. solani* (right). Photographs were taken 10 days after inoculation. (C) Confrontation against *R. solani* by the secretion of extracellular enzymes from *T. harzianum*. *Trichoderma* strains were cultured on cellophane in PDA agar plates at 28°C for 48 h. The cellophane was removed and the plates were re-inoculated with *R. solani* for 3 days. (D) The growth rate of *R. solani* was calculated. Different letters indicate statistically significant differences (*P*≤0.01).

We observed no significant difference in the ability of *T. harzianum* T28 or KO40 to overgrow and conidiate against *C. lunata*, *B. cinerea*, or *F. graminearum* (Fig. 7A). However, KO40 failed to overgrow *R. solani* compared with wild type strain (Fig. 7B). To investigate the mechanism of reduced antagonistic ability in KO40, we conducted a secretion assay in which *R. solani* was grown on agar plates previously colonized by T28 or KO40 strains to detect its sensitivity to *T. harzianum* secreted factors. Results showed the *R. solani* growth rate was notably lower (*P*<0.01) on agar plates previously colonized by *T. harzianum* but was higher (*P*<0.01) on agar plates previously colonized by KO40 (Fig. 7C and 7D). Hence, the results suggest PAF-AH plays a vital role in the antagonistic activity of *T. harzianum* against *R. solani*.

## Discussion

This work investigated the important role of PAF-AH, a member of the enzyme superfamily PLA_2_, in *T. harzianum* physiology, stress response, and antagonism. The involvement of eicosanoids and leukotrienes in inflammatory mediation has been extensively demonstrated [2], [30], [31], and PAF-AH hydrolyzes phospholipids into the precursors of these molecules. In this study, we purified PAF-AH derived from *T. harzianum* and explored its biochemical characteristics *in vitro*. The PAF-AH gene was amplified from genomic DNA of *T. harzianum* T28 and was found to share 99% and 70% of similarity with PAF-AH from *T. koningii* (Accession no. ACQ83643.1) and *Cordyceps militaris* (Accession no. EGX90692.1), respectively (Fig. 1).

We also analyzed PAF-AH in *T. harzianum*'s fatty acid and sterol profiles by GC/MS. The results not only revealed the hydrolytic mechanism of PAF-AH but also indicated PAF-AH promotes stress response associated with increased levels of eicosanoid and ergosterol. Other studies have previously reported that eicosanoid and ergosterol are involved with stress response [32], [33]. Specifically, Moretti-Almeida, et al. [34] has suggested ergosterol biosynthesis plays an important role in maintaining mitochondrial and plasma membrane integrity and mediating an antioxidant response. The integrity of cytoplasmic and mitochondrial membranes are associated with ergosterol, and increased levels of unsaturated fatty acids and the production of ergosterol occurs during chemical [35] and temperature stress in *S. cerevisiae* [36]; in both cases, the increased ergosterol levels conferred stress tolerance to *S. cerevisiae*. Interestingly, in this work, we observed PAF-AH-mediated thermotolerance in *T. harzianum* with concomitant upregulation of PAF-AH mRNA expression. We hypothesized PAF-AH might be associated with cytosolic proteins to regulate ergosterol production to maintain plasma membrane integrity, and the exact regulation mechanism of PAF-AH to change the production of ergosterol will be explored in a future study.

**Figure 8:**
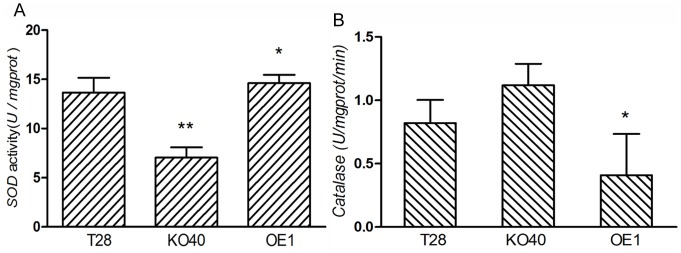
SOD and catalase activity assay of strains T28, KO40, and OE1 (A). Catalase activity assay of T28, KO40, and OE1 (B). The experiment was carried out with the same amount of tissue protein dissolved in 0.1 M PBS. * indicated significant differences between enzyme activity compared with the wild type (Student's *t*-test, *P*≤0.05), and * * represents *P*≤0.01. Results shown are means ± SD (n = 3).

To understand PAF-AH's functions related to stress response in *T. harzianum*, we used 2-DE analysis to compare differentially expressed protein spots between wild type and KO40 transformant strains. Fifteen differentially expressed protein spots were observed, and of these, six were upregulated and nine were downregulated. Interestingly, expression of HEX1, Cu/Zn SOD and cytochrome c was downregulated in transformant KO40, suggesting PAF-AH positively regulates proteins involved with stress resistance. Similarly, the protein HEX1 in *T. atroviride* promotes tolerance of organophosphate pesticide accumulation *in vitro* [12], and superoxide dismutases (SODs) are vital metallo-enzymes involved in cellular protection against superoxide [37]. SOD activity was lower in transformant KO40 compared with the wild type but higher in the PAF-AH over-expression strain OE1 (Fig. 8A). We observed higher catalase activity in KO40 than in the wild-type (Fig. 8B), likely due to reduced expression of Cu/Zn SOD and accumulation of reactive oxygen species due to decreased PAF-AH activity. The 2-DE assay did not identify any differentially expressed proteins or related products of ergosterol.

*Trichoderma* has a strong capability to overwhelmingly outcompete rival organisms for nutrients and space during stress conditions [8]. We observed significantly varied growth rates among strains T28, KO40, and OE1 at a pH range of 3–10; for example, the growth rate of KO40 was slower than T28 and OE1 at pH 8 and pH 10 (Fig. S4). The growth performances of the strains were not significantly different at 5 mM and 10 mM H_2_O_2_ on SM agar plates, but the OE1 strain grew well on the 50 mM H_2_O_2_ SM agar plate (Fig. S5).

We also determined PAF-AH is necessary for the antagonistic ability of *T. harzianum* against *R. solani* in plate confrontation assays. Expression of the chitinase gene *chit42* was downregulated in KO40 (data not shown), possibly due to reduced secreted enzymes that are important to *T. harzianum*'s mycoparasitic interactions with *R. solani*. Another reason for reduced antagonism of KO40 may be related to deficient PAF-AH-induced sensitivity to *R. solani*-secreted enzymes or secondary metabolic products that affect cell membrane integrity of *Trichoderma*.

*Trichoderma* spp. exhibit remarkable features, such as fast growth, colonization, and efficient utilization of nutrients in the environment. These characteristics are common in fungi; for instance, the mRNA of TbSP1 (an important gene from phospholipases A2) of *T. borchii* is significantly upregulated when carbon or nitrogen sources are scarce, and the expression of two other PLA_2_ mRNAs is elevated in *Aspergillus oryzae* under carbon starvation and oxidative stress conditions [38], [39]. Similarly, our present work also found PAF-AH mRNA in *T. harzianum* was notably upregulated in response to carbon source starvation and temperature stress. Furthermore, we determined interactions between *T. harzianum* and maize roots led to increased PAF-AH expression. Because PAF-AH might interact with maize seedling roots in rhizosphere, during which nitrogen and carbon supplies may be limited, we propose increased PAF-AH activity would favor *Trichoderma* growth under carbon starvation conditions in barren soil.

In summary, this present work was the first report regarding the biological role of PAF-AH in *T. harzianum* stress response and antagonism. This protein mediates tolerance to abiotic stress and the microbe's antagonistic ability toward *R. solani*. Additionally, PAF-AH might be involved and in scarce nutrient utilization of *Trichoderma* and interactions with maize seedling roots in the rhizosphere. We conclude PAF-AH has versatile function in *Trichoderma*, and its gene expression is likely triggered at different environments to aid in *Trichoderma*'s adaptation and survival.

## Supporting Information

Figure S1 **Screening of PAF-AH overexpression transformants.** (A) Physical map of overexpression cassette. (B) Amplified fragment of *Hygromycin B* gene. (C) Amplified fragment of promoter *trp C*. (D) Amplified fragment of PAF-AH. (E) Amplified fragment of terminator *trp C*. (F) Amplified fragment of PAF-AH overexpression cassette. (G) Southern blot to confirm PAF-AH overexpression transformants. The genome DNA of WT and transformants were digested with *Xba* I. The 450 bp fragment amplified from *Hygromycin B* gene was labeled with ^32^P-dCTP as probe.WT: wild-type strain. P: plasmid. 1, 2, 3: PAF-AH overexpression transformants. M: DNA maker.
(TIF)

Figure S2 **PAF-AH transcription level analysis in KO15 and KO40 transformants.** (A) Electrophoresis of PCR products of PAF-AH ORF. (B) Electrophoresis of PCR products of 500 bp fragment of the PAF-AH. The PCR template was 1 µL cDNA of WT T28 and the KO transformants respectively. M: DNA marker, WT: Wild Type (T28), KO15, KO40: PAF-AH KO transformants.
(TIF)

Figure S3 **PAF-AH activity assay of T28, KO40, and OE1.** The experiment was carried out with the same amount of tissue protein dissolved in 0.1 M PBS, and these strains were cultured the same as in 2-DE protein extraction. Data were measured with Microplate Reader. * indicated significant difference of enzyme activity compared with the wild type, * showed *P*≤0.05. Results were means±SD (n = 3).
(TIF)

Figure S4 **Different strains grew on different pH SM plate agar.** The photograph was taken after 24 h inoculation at 28°C, T28 (wild type), KO40 (PAF-AH KO transformant), OE1 (PAF-AH overexpression transformant).
(TIF)

Figure S5 **Different strains grew on SM plate agar containing different concentration of H_2_O_2_.** The photograph was taken after 24 h inoculation at 28°C, T28 (wild type), KO40 (PAF-AH KO transformant), OE1 (PAF-AH overexpression transformant).
(TIF)

Table S1 **Primer used in PCR reaction of this paper.**
(DOC)

Table S2 **qRT-PCR efficiency of PAF-AH at different condition.**
(DOC)

Table S3 **qRT-PCR efficiency of** ***hex1*** **,** ***cu/zn sod*** **,** ***cytochrome c*** **.**
(DOC)
