# Supplementary figures and images for: Biological Role of Trichoderma harzianum-Derived Platelet-Activating Factor Acetylhydrolase (PAF-AH) on Stress Response and Antagonism
Source: PLoS One. 2014 Jun 25;9(6):e100367. doi: 10.1371/journal.pone.0100367 (PMC4070952; doi:10.1371/journal.pone.0100367)

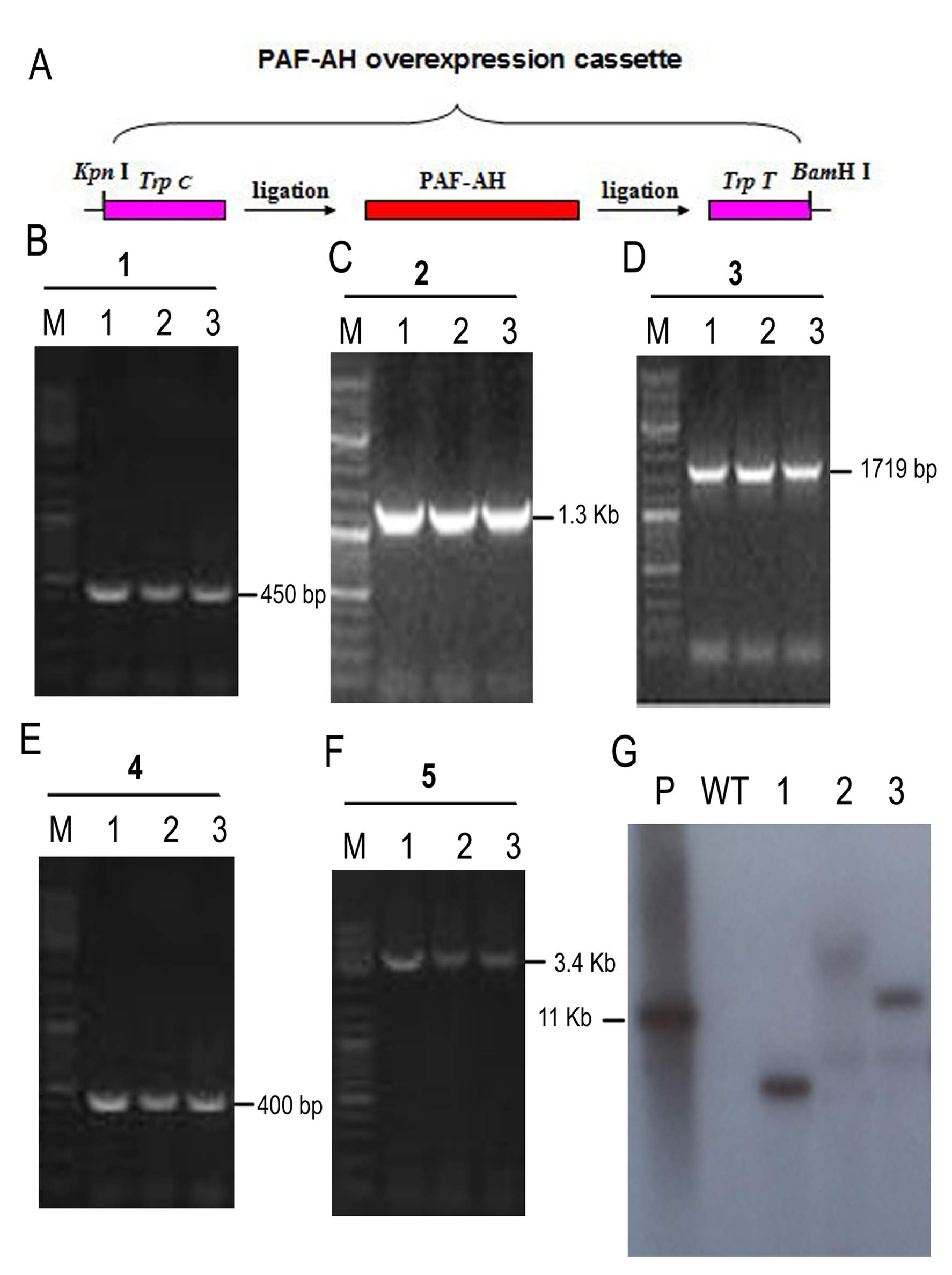

Supplement: Figure S1 — Screening of PAF-AH overexpression transformants. (A) Physical map of overexpression cassette. (B) Amplified fragment of Hygromycin B gene. (C) Amplified fragment of promoter trp C. (D) Amplified fragment of PAF-AH. (E) Amplified fragment of terminator trp C. (F) Amplified fragment of PAF-AH overexpression cassette. (G) Southern blot to confirm PAF-AH overexpression transformants. The genome DNA of WT and transformants were digested with Xba I. The 450 bp fragment amplified from Hygromycin B gene was labeled with 32P-dCTP as probe.WT: wild-type strain. P: plasmid. 1, 2, 3: PAF-AH overexpression transformants. M: DNA maker. (TIF) [file pone.0100367.s001.tif]

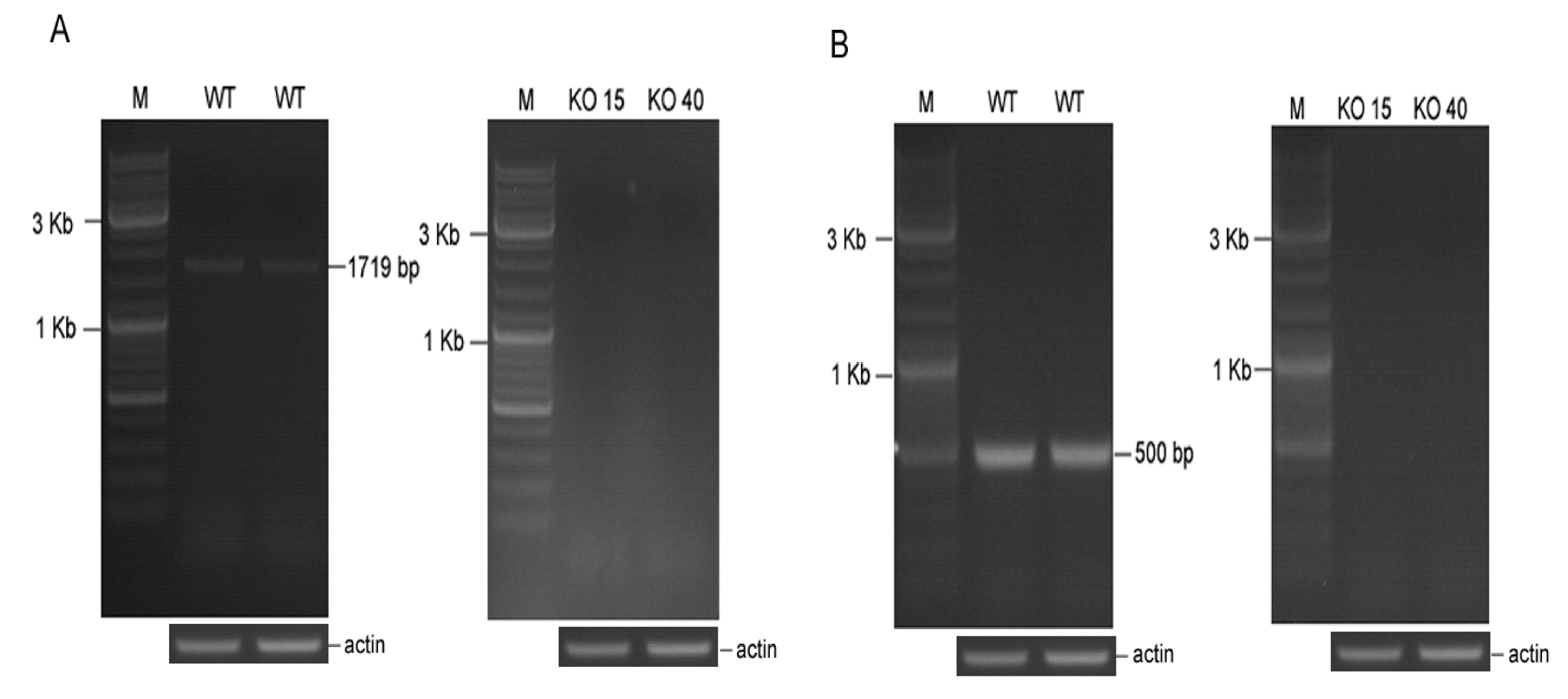

Supplement: Figure S2 — PAF-AH transcription level analysis in KO15 and KO40 transformants. (A) Electrophoresis of PCR products of PAF-AH ORF. (B) Electrophoresis of PCR products of 500 bp fragment of the PAF-AH. The PCR template was 1 µL cDNA of WT T28 and the KO transformants respectively. M: DNA marker, WT: Wild Type (T28), KO15, KO40: PAF-AH KO transformants. (TIF) [file pone.0100367.s002.tif]

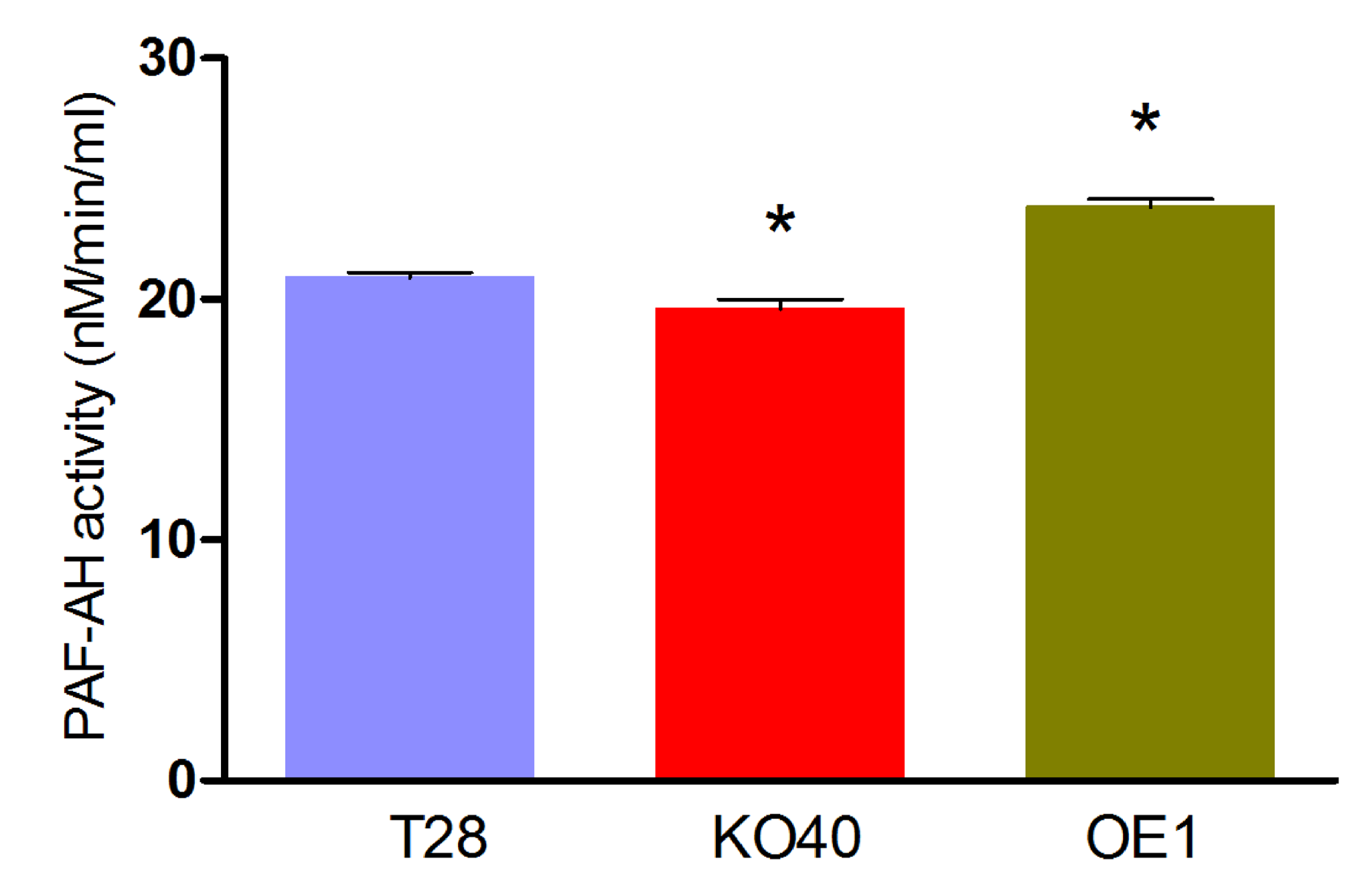

Supplement: Figure S3 — PAF-AH activity assay of T28, KO40, and OE1. The experiment was carried out with the same amount of tissue protein dissolved in 0.1 M PBS, and these strains were cultured the same as in 2-DE protein extraction. Data were measured with Microplate Reader. * indicated significant difference of enzyme activity compared with the wild type, * showed P≤0.05. Results were means±SD (n = 3). (TIF) [file pone.0100367.s003.tif]

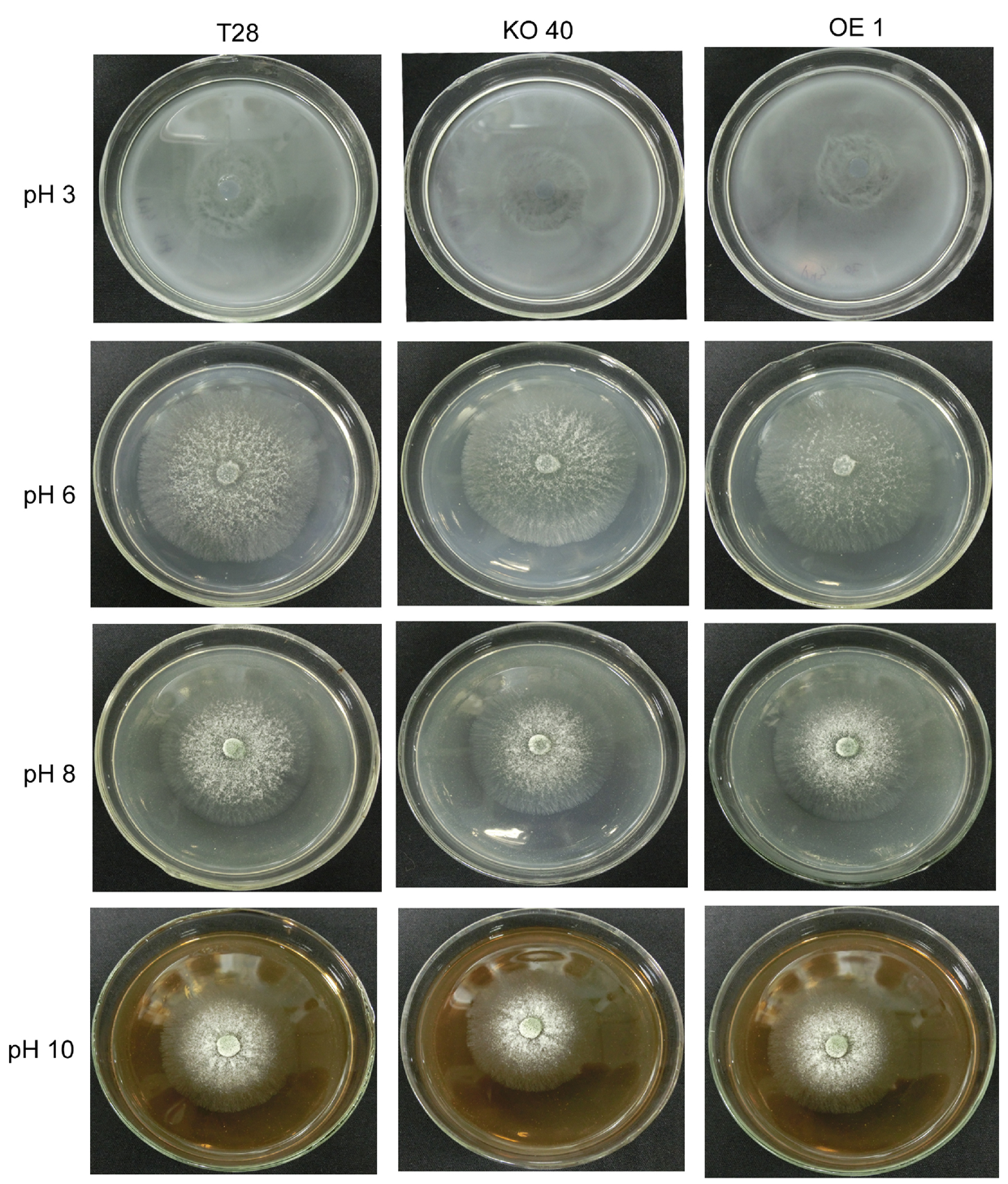

Supplement: Figure S4 — Different strains grew on different pH SM plate agar. The photograph was taken after 24 h inoculation at 28°C, T28 (wild type), KO40 (PAF-AH KO transformant), OE1 (PAF-AH overexpression transformant). (TIF) [file pone.0100367.s004.tif]

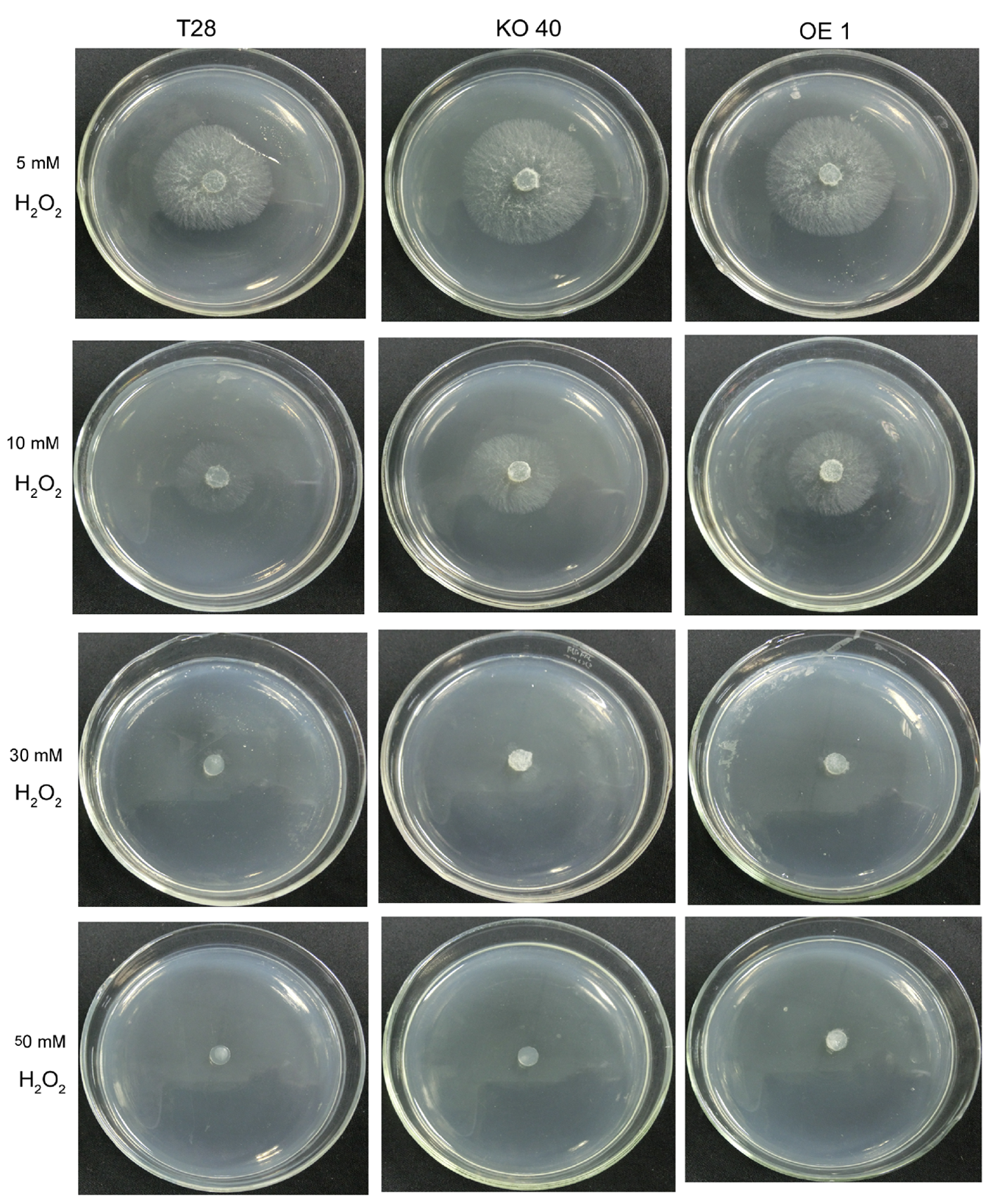

Supplement: Figure S5 — Different strains grew on SM plate agar containing different concentration of H2O2. The photograph was taken after 24 h inoculation at 28°C, T28 (wild type), KO40 (PAF-AH KO transformant), OE1 (PAF-AH overexpression transformant). (TIF) [file pone.0100367.s005.tif]
