# Supplementary material for: Biological Role of Trichoderma harzianum-Derived Platelet-Activating Factor Acetylhydrolase (PAF-AH) on Stress Response and Antagonism
Source: PLoS One. 2014 Jun 25;9(6):e100367. doi: 10.1371/journal.pone.0100367 (PMC4070952; doi:10.1371/journal.pone.0100367)
Supplement: Table S1 — Primer used in PCR reaction of this paper. (DOC) [file pone.0100367.s006.doc]

**Table S1.** Primer used in PCR reaction of this paper

| Primers | Sequence (5’ to 3’) |
| --- | --- |
| KO-up-F | GCAAGCTTACGCCAGTTCACCTTAGCATTTGT |
| KO- up -R | TATCTAGAGGTGGCGATTGCGATTGCGACGGCGA |
| KO-down-F | GTGGTACCTCTCACGGCTGCTCAACAGCATT |
| KO-down-R | GTGAGCTCAGTGGCAGGGTTCTTCAAGGGAGAT |
| Hyg-F | GCTCTCGGAGGGCGAAGAAT |
| Hyg-R | GCCTGCGCGACGGACGCACTG |
| RB-F | GATCCAAGCTCAAGCTGCTCT |
| PAF-AH-UP-F | ACACACGGCCTCTTCCCAGA |
| PAF-AH-F | TGAGAATTCATGCTTGGCGCTTCAGAGC |
| PAF-AH-R | GGAGGATCCTTAAATCTCCTGCCAGCC |
| Probe PAF-AH-F | GTGACGGAGAAGAATGCCAC |
| Probe PAF-AH-R | GCCAAGTCCAGCTTGTCCAT |
| hex1-F | GTGAATTCATGGGTTACTACGACGAC |
| hex1-R | GTGGATCCTTACAGGCGAGAGCCGTGGAC |
| cu/zn sod -F | GTGAATTCATGGTCAAAGCCGTCACTGT |
| cu/zn sod -R | GTGGATCCTTAAGCAGAGATGCCGAT |
| cytochrome c-F | GTGAATTCATGGGTTTCTCTGAGGGC |
| cytochrome c-R | GTGGATCCTTATTTGGTGTTCTCCTTG |
| qPAF-AH-F | ACACGACGTCATCGACCATT |
| qPAF-AH-R | TTCACTGGGCTCTAACTCGC |
| q hex1-F | TGTCAAGCAGGGTCTGTCTG |
| q hex1-F | CGTGGACGACCTTCATCTCAA |
| q cu/zn sod -F | GATGCTCAGGGTAACGCCAA |
| q cu/zn sod -R | TTGCCAGTCTTGAGGGACTC |
| q cytochrome c -F | GACAAGACCCTCTTCGCCTAC |
| q cytochrome c -R | TATTTGGTAGCGTCCTTGAGGTA |
| qT. harzianum actin-F | GTATCATGATCGGTATGGGTCAGA |
| qT. harzianum actin-R | TAGAAGGTGTGGTGCCAGATCTT |
| Walking SP primers1  Walking SP primers2  Walking SP primers3 | AACGAGATCTCCAATCCAAGTC  GGGCTCCGGAAGCTTGTTGCCA  GTTGAACAGAGGAGTCGAGTTG |
