# Supplementary material for: Biological Role of Trichoderma harzianum-Derived Platelet-Activating Factor Acetylhydrolase (PAF-AH) on Stress Response and Antagonism
Source: PLoS One. 2014 Jun 25;9(6):e100367. doi: 10.1371/journal.pone.0100367 (PMC4070952; doi:10.1371/journal.pone.0100367)
Supplement: Table S2 — qRT-PCR efficiency of PAF-AH at different condition. (DOC) [file pone.0100367.s007.doc]

**Table S2.** qRT-PCR efficiency of PAF-AH at different condition

|  | PAF-AH |  |  | PAF-AH |  |
| --- | --- | --- | --- | --- | --- |
| Treatment alone | Ct | PCR efficiency | + Maize root | Ct | PCR efficiency |
| SM | 24.4333 | 1.4884 | SM | 24.6201 | 1.5308 |
|  | 24.92 | 1.6124 |  | 24.4312 | 1.5613 |
|  | 24.5975 | 1.5218 |  | 24.4978 | 1.483 |
|  | 24.4401 | 1.5152 |  | 24.6013 | 1.4941 |
|  | 24.893 | 1.6075 |  | 24.4297 | 1.578 |
|  | 24.5894 | 1.4908 |  | 24.5237 | 1.563 |
|  | 24.4245 | 1.5123 |  | 24.6131 | 1.4704 |
|  | 24.9087 | 1.6076 |  | 24.4052 | 1.5146 |
|  | 24.6081 | 1.5801 |  | 24.5307 | 1.5287 |
| PDA | 24.6872 | 1.6883 | -C | 24.865 | 1.5372 |
|  | 24.4855 | 1.6522 |  | 24.8672 | 1.6154 |
|  | 24.6777 | 1.5264 |  | 24.9411 | 1.6055 |
|  | 24.5987 | 1.4984 |  | 24.865 | 1.5735 |
|  | 24.5514 | 1.6116 |  | 24.8704 | 1.6308 |
|  | 24.6488 | 1.5872 |  | 24.9244 | 1.5938 |
|  | 24.6795 | 1.6607 |  | 24.8375 | 1.5206 |
|  | 24.4834 | 1.5896 |  | 24.8875 | 1.4988 |
|  | 24.6801 | 1.5707 |  | 24.9503 | 1.6564 |
| -C | 24.3236 | 1.6423 | -N | 24.0446 | 1.5803 |
|  | 24.5009 | 1.5131 |  | 23.8053 | 1.6534 |
|  | 24.2353 | 1.6733 |  | 23.5385 | 1.6482 |
|  | 24.3132 | 1.6056 |  | 24.1341 | 1.5876 |
|  | 24.4875 | 1.4891 |  | 23.9766 | 1.4908 |
|  | 24.2178 | 1.5367 |  | 23.5865 | 1.6836 |
|  | 24.3401 | 1.6152 |  | 24.0997 | 1.5709 |
|  | 24.5101 | 1.5341 |  | 23.8106 | 1.6174 |
|  | 24.2402 | 1.6089 |  | 23.5378 | 1.6388 |
| -N | 24.8298 | 1.62 | -CN | 23.0789 | 1.6518 |
|  | 24.9643 | 1.5261 |  | 23.5361 | 1.6302 |
|  | 25.1229 | 1.5436 |  | 24.0042 | 1.7609 |
|  | 24.8166 | 1.5773 |  | 23.0835 | 1.6602 |
|  | 24.9553 | 1.4788 |  | 23.5411 | 1.6115 |
|  | 25.1378 | 1.5646 |  | 24.1009 | 1.4992 |
|  | 24.8245 | 1.6019 |  | 23.1103 | 1.5924 |
|  | 24.9499 | 1.5802 |  | 23.5775 | 1.6211 |
|  | 25.2089 | 1.5166 |  | 23.9987 | 1.6046 |
| -CN | 25.8114 | 1.5975 | 4 °C | 24.0845 | 1.4932 |
|  | 25.5552 | 1.6126 |  | 24.3867 | 1.5317 |
|  | 25.5532 | 1.6435 |  | 24.0829 | 1.4813 |
|  | 25.8214 | 1.5422 |  | 24.0933 | 1.5266 |
|  | 25.5467 | 1.6089 |  | 24.3912 | 1.6805 |
|  | 25.5138 | 1.6035 |  | 24.1102 | 1.4968 |
|  | 25.8098 | 1.4622 |  | 24.0799 | 1.5506 |
|  | 25.5368 | 1.6641 |  | 24.3913 | 1.6144 |
|  | 25.5611 | 1.6235 |  | 24.0617 | 1.5302 |
| 4 °C | 24.9681 | 1.5734 | 40°C | 24.8214 | 1.6751 |
|  | 24.2185 | 1.5421 |  | 25.5745 | 1.5561 |
|  | 25.6238 | 1.4974 |  | 24.768 | 1.5805 |
|  | 24.9533 | 1.5536 |  | 24.8321 | 1.4751 |
|  | 24.2209 | 1.5788 |  | 25.5688 | 1.5984 |
|  | 25.6153 | 1.6036 |  | 24.7597 | 1.5754 |
|  | 24.8991 | 1.4734 |  | 24.8276 | 1.6106 |
|  | 24.2148 | 1.5178 |  | 25.5802 | 1.5778 |
|  | 25.6308 | 1.5504 |  | 24.7512 | 1.5467 |
| 40°C | 23.9817 | 1.546 |  |  |  |
|  | 23.8456 | 1.5341 |  |  |  |
|  | 23.8129 | 1.672 |  |  |  |
|  | 23.9788 | 1.6058 |  |  |  |
|  | 23.8312 | 1.4711 |  |  |  |
|  | 23.826 | 1.6399 |  |  |  |
|  | 23.9834 | 1.5529 |  |  |  |
|  | 23.8373 | 1.5324 |  |  |  |
|  | 23.8087 | 1.6287 |  |  |  |
| H_2_O_2_ | 25.021 | 1.5503 |  |  |  |
|  | 24.8582 | 1.5215 |  |  |  |
|  | 23.5907 | 1.6102 |  |  |  |
|  | 25.1302 | 1.5175 |  |  |  |
|  | 24.7316 | 1.5743 |  |  |  |
|  | 23.6142 | 1.602 |  |  |  |
|  | 24.998 | 1.4822 |  |  |  |
|  | 24.8887 | 1.5869 |  |  |  |
|  | 23.4401 | 1.6605 |  |  |  |
