# Supplementary material for: Biological Role of Trichoderma harzianum-Derived Platelet-Activating Factor Acetylhydrolase (PAF-AH) on Stress Response and Antagonism
Source: PLoS One. 2014 Jun 25;9(6):e100367. doi: 10.1371/journal.pone.0100367 (PMC4070952; doi:10.1371/journal.pone.0100367)
Supplement: Table S3 — qRT-PCR efficiency of hex1 , cu/zn sod , cytochrome c . (DOC) [file pone.0100367.s008.doc]

**Table S3.** qRT-PCR efficiency of *hex1*, *cu/zn sod*, *cytochrome c*

|  | *hex1* |  | *hex1* |  | *cu/zn sod* |  | *cu/zn sod* |  | *cytochrome c* |  | *cytochrome c* |
| --- | --- | --- | --- | --- | --- | --- | --- | --- | --- | --- | --- |
| T28 | PCR | KO40 | PCR | T28 | PCR | KO40 | PCR | T28 | PCR | KO40 | PCR |
| Ct | efficiency | Ct | efficiency | Ct | efficiency | Ct | efficiency | Ct | efficiency | Ct | efficiency |
| 18.9931 | 1.6827 | 18.6837 | 1.7272 | 24.2464 | 1.7136 | 23.9016 | 1.6902 | 21.2126 | 1.7318 | 21.1766 | 1.7403 |
| 19.27 | 1.7134 | 18.7282 | 1.7025 | 24.2408 | 1.7544 | 23.8924 | 1.7601 | 21.3882 | 1.7674 | 21.3285 | 1.6962 |
| 19.2883 | 1.7978 | 18.5928 | 1.7132 | 24.3993 | 1.7890 | 23.8371 | 1.6550 | 21.6091 | 1.6751 | 21.3161 | 1.7590 |
| 18.984 | 1.7013 | 18.598 | 1.7014 | 24.2502 | 1.6823 | 23.9101 | 1.6891 | 21.2098 | 1.6952 | 21.2103 | 1.7024 |
| 19.19 | 1.6724 | 18.746 | 1.6796 | 24.239 | 1.7601 | 23.8798 | 1.7206 | 21.3743 | 1.7203 | 21.3197 | 1.6907 |
| 19.212 | 1.7202 | 18.583 | 1.7204 | 24.387 | 1.6873 | 23.8654 | 1.6937 | 21.6105 | 1.6877 | 21.3209 | 1.6892 |
| 18.995 | 1.6894 | 18.713 | 1.6798 | 24.3881 | 1.6908 | 23.889 | 1.7102 | 21.2188 | 1.6938 | 21.2704 | 1.7109 |
| 19.314 | 1.7047 | 18.705 | 1.7806 | 24.2608 | 1.7086 | 23.9073 | 1.6887 | 21.3903 | 1.7305 | 21.2997 | 1.6935 |
| 19.281 | 1.6989 | 18.602 | 1.6908 | 24.3693 | 1.6899 | 23.8491 | 1.7011 | 21.5986 | 1.6741 | 21.2471 | 1.7301 |
